# Resolutions of the Cleveland Dental Society

**Published:** 1893-04

**Authors:** 


### Resolutions of the Cleveland Dental Society.

The Cleveland Dental Society hereby express their sympathy to the family of Dr. Geo. Watt, of Xenia, Ohio:

*Resolved*, That, in the death of Dr. Watt, the dental profession has met with a severe loss. His talents were for our use, and his gifts were numerous. The inspiration of such a life will lead other men to usefulness. The memory of Dr. Watt's work will live though he has passed to a better reward beyond this earthly existence.

We honor him.

H. L. Ambler,
W. H. Whitslar,
S. B. Dewey.
Committee.

